# Persistence of arctic-alpine flora during 24,000 years of environmental change in the Polar Urals

**DOI:** 10.1038/s41598-019-55989-9

**Published:** 2019-12-23

**Authors:** C. L. Clarke, M. E. Edwards, L. Gielly, D. Ehrich, P. D. M. Hughes, L. M. Morozova, H. Haflidason, J. Mangerud, J. I. Svendsen, I. G. Alsos

**Affiliations:** 1 School of Geography and Environmental Science, University of Southampton, Highfield, Southampton, SO17 1BJ UK; 2 Laboratoire d’Ecologie Alpine (LECA), Université Grenoble Alpes, C2 40700 38058 Grenoble, Cedex 9 France; 3 Department of Arctic and Marine Biology, UiT- The Arctic University of Norway, Tromsø, NO-9037 Norway; 4 Institute of Plant and Animal Ecology, Ural Branch of Russian Academy of Sciences, Ekaterinburg, Russia; 5 Department of Earth Science and Bjerknes Centre for Climate Research, University of Bergen, Allégaten 41, Bergen, 5007 Norway; 6 Tromsø University Museum, UiT - The Arctic University of Norway, NO-9037 Tromsø, Norway

**Keywords:** Conservation biology, Palaeoecology

## Abstract

Plants adapted to extreme conditions can be at high risk from climate change; arctic-alpine plants, in particular, could “run out of space” as they are out-competed by expansion of woody vegetation. Mountain regions could potentially provide safe sites for arctic-alpine plants in a warmer climate, but empirical evidence is fragmentary. Here we present a 24,000-year record of species persistence based on sedimentary ancient DNA (*sed*aDNA) from Lake Bolshoye Shchuchye (Polar Urals). We provide robust evidence of long-term persistence of arctic-alpine plants through large-magnitude climate changes but document a decline in their diversity during a past expansion of woody vegetation. Nevertheless, most of the plants that were present during the last glacial interval, including all of the arctic-alpines, are still found in the region today. This underlines the conservation significance of mountain landscapes via their provision of a range of habitats that confer resilience to climate change, particularly for arctic-alpine taxa.

## Introduction

Arctic-alpine plants are considered to be at greater risk of habitat loss and local extinction under future climate change than plants of lower elevations^1–3^. Yet model simulations and predictions at larger scales often fail to account for the importance of local-scale factors that control plant distributions^4,5^, and it may be that extinction probabilities have been overestimated. Indeed, observed extinction rates on mountain tops have been low, despite climate warming over the past century^6,7^. This uncertainty underlines the importance of identifying specific geographical locations and/or attributes of habitats that have helped to sustain communities over long periods of varying climate and documenting their long-term history. Recent advances in sedimentary ancient DNA (*sed*aDNA) analysis (see below), when combined with a high-quality sediment record, promise to provide new insights into the diversity of the arctic-alpine flora and how it has fared through large-magnitude climate changes in the past. Therefore, we collected data on species persistence using *sed*aDNA analysis on a high-resolution and well-studied lake sediment core spanning the last 24,000 years from the Polar Ural Mountains of the Russian Arctic.

Areas that facilitated long-term species persistence in the past can be considered a priority for conserving biodiversity and genetic diversity under a changing climate^8^. Spatially heterogeneous mountain landscapes should provide viable future habitat for taxa with a range of environmental requirements. In such landscapes, microclimate and soil vary with topography and elevation to create a mosaic of different conditions at the mesoscale^9^, thus providing a buffer against regional climate changes^10–12^. At the same time, compressed vertical and horizontal gradients enable species to track their bioclimatic niches effectively as climate changes^13,14^. Most evidence that mountain regions function as long-term refugia or safe sites is, however, indirect, being based on contemporary observation and/or modelling^3,5,15^.

With future warming exacerbated by arctic amplification^16,17^, much terrain in northern lowlands may be taken over by woody plant communities^18–20^, making northern mountain areas important as localities in which competition-sensitive arctic-alpines can persist. While we have some knowledge about long-term refugia for warm-adapted species during cold, glacial periods^21^, identifying locations for arctic-alpine species in warm, interglacial periods has received much less attention. Identifying such locations requires a challenging integration of long temporal timescales and fine spatial scales^22^. Fossils (i.e. pollen/plant macrofossils) provide the best evidence for presence of a plant species at a given time in the past, but records can be constrained by several factors: levels of preservation and taxonomic resolution, specific nature of the site, etc.^23–25^. Previous studies have often had to rely on interpolation and/or extrapolation to argue for long-term species persistence, based on sporadic occurrences of a taxon in the fossil record and its presence within the present-day vegetation mosaic^26–28^.

Analysis of *sed*aDNA has the potential to provide more detail on past community composition, particularly with regard to arctic-alpine species, as the method is well suited to cold climates^29–31^ and local, well-curated plant reference libraries are available from northern regions^29,32,33^. It can significantly augment information on plant community composition and species persistence derived from traditional fossil records^34–36^. In contrast to pollen, the *sed*aDNA signal is less sensitive to “swamping” by woody anemophilous taxa (e.g. many north-temperate and boreal trees and shrubs) at the expense of taxa that have poor pollen representation—such as insect-pollinated arctic-alpine herbs^37–39^.

**Figure 1:**
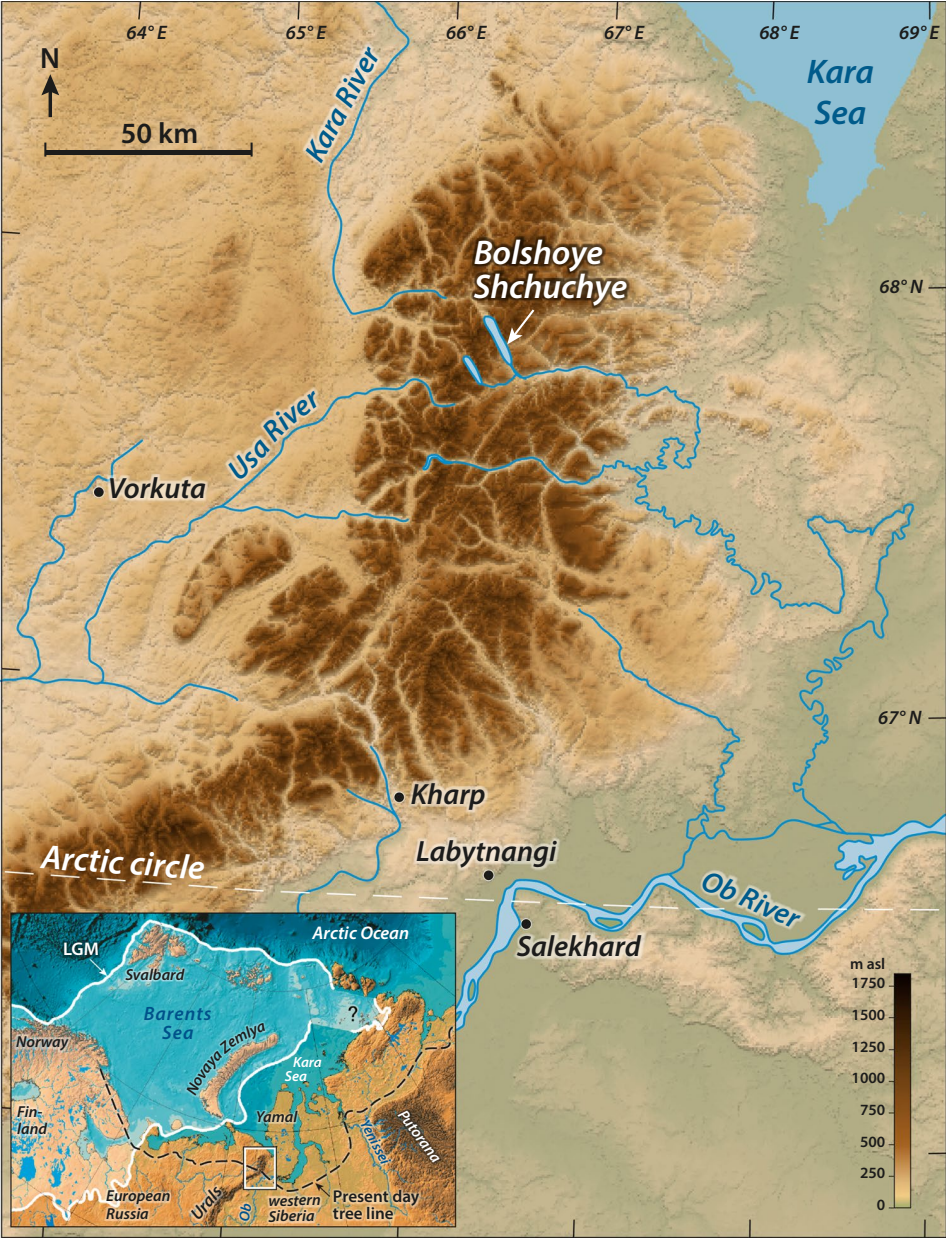
Location of the study area. Digital elevation model of the Polar Urals with the location of Lake Bolshoye Shchuchye marked. Map inset shows the location (rectangle) of main map. The ice sheet limit during the Last Glacial Maximum (white line) from Svendsen *et al*. (2004) and the present-day boreal treeline (black dashed line) are also indicated. Map generated using ESRI ArcMap 10.5.1 (http://desktop.arcgis.com/en/arcmap/).

Here we present a continuous 24,000-year record of plant community composition and species persistence based on ancient DNA extracted from lake sediments (*sed*aDNA). We use the well-described sediments of Lake Bolshoye Shchuchye^40–42^, the largest and deepest lake in the Polar Ural Mountains (Fig. 1). The sediment record is unique for western Eurasia as it represents 24,000 years without any breaks and/or disturbances. The site has remained ice free for at least the last 60,000 years, thus providing insight into a well-established and diverse flora—compared with adjacent regions that were deglaciated after the Last Glacial Maximum (LGM). Moreover, the lake catchment supported the establishment of forest at lower elevations for a period in the Holocene between ca. 9000 and 4000 cal. years BP. Compared with the smaller lakes typically used for reconstructions of vegetation histories based on *sed*aDNA^35,43–45^, Lake Bolshoye Shchuchye’s sediments may capture a signal of plant communities over an elevational range of 187–1,100 m a.s.l. within its large (215 km^2^) hydrologic catchment. The lake is surrounded by steeply sloping hillsides and is fed by the River Pyriatanyu, which drains much of the catchment; this means that the lake probably receives direct local slope-wash plus fluvially transported material from a much wider area. The lake catchment is physiographically diverse and thus answers a key criterion suggested by modelling studies to facilitate long-term species persistence. We use metabarcoding techniques^46^ to establish the *sed*aDNA record and combine the results with information on the present-day occurrence of identified taxa within the catchment vegetation mosaic.

Our main aim is to test the degree of persistence of elements of the flora in the catchment of Lake Bolshoye Shchuchye over the past 24,000 years, with a particular focus on the fate of competition-sensitive, arctic-alpine plants during the past expansion of woody taxa. The *sed*aDNA record provides insight into long-term preservation of floristic diversity at the catchment scale in a mountain landscape, and it documents the response of individual arctic-alpine plants to expanding woody vegetation. Results show the catchment has supported plant communities that have diversified through time; these include typical arctic-alpine assemblages and also taxa of shrub tundra and boreal forest. We conclude that this spatially heterogeneous mountain landscape has evidently functioned effectively as a refugium for cold-adapted plants in a warm climate.

## Results

### Plant sedaDNA record

We obtained around  75 million paired-end raw DNA sequences for the 153 sediment samples from Lake Bolshoye Shchuchye (Supplementary Table S1). After filtering out sequencing artefacts and sequences with <98% match to the DNA reference library (see Methods), we retained 19 million reads, corresponding to 134 vascular plant and 28 bryophyte taxa. Of these, 40% are identified to species level, 45% to genus and 15% to a higher taxonomical level (Supplementary Table S2). Where possible, genera that were not identified to species level were assigned to a likely inferred species based on their biogeographic distribution (Supplementary Table S2).

The identified taxa represent a wide range of different modern ecological habitats, including forest (e.g. *Larix sibirica*, *Picea* sp.) and its understory (e.g. *Dryopteris fragrans*, *Gymnocarpium dryopteris)*, tall shrubs (e.g. *Alnus*, *Betula*, *Salix*), dwarf shrub-tundra (e.g. *Vaccinium uligonosum*, *V. vitis-idaea/myrtillus, Arctostaphylos uva-ursi*, *Empetrum nigrum, Dryas octopetala)* herb-tundra (e.g. *Puccinellia*, *Festuca*, *Draba*, *Lagotis glauca*), mesic sedge (e.g. *Carex*, *Eriophorum*) and bryophyte (e.g. *Andreaea*, *Aulacomnium turgidum*, Dicranaceae) communities, and (in all probability) mixed plant communities in between these categories (Supplementary Figs. S1–S3).

**Figure 2:**
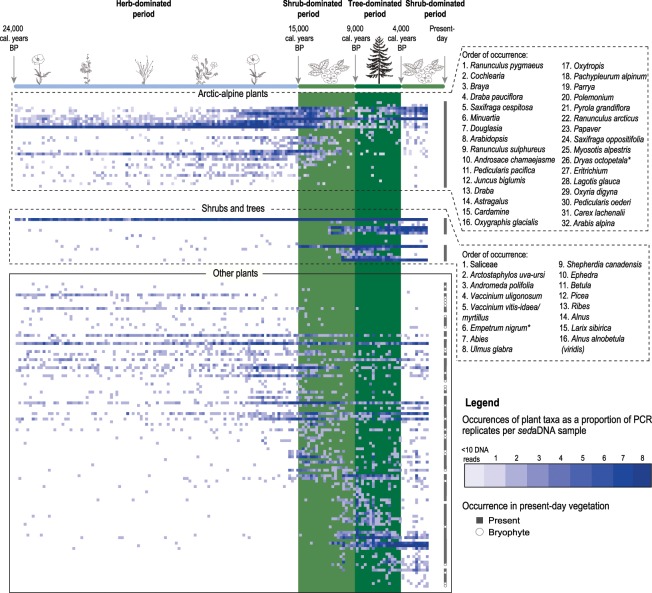
Incidence matrix of plant taxa detected in Lake Bolshoye Shchuchye’s sediments using *sed*aDNA analysis. All identified plant taxa are presented as a proportion of PCR replicates (out of eight) per *sed*aDNA sample. Plant taxa classified as arctic-alpine and shrub and tree taxa are highlighted within separate dashed areas and taxon names are given in order of occurrence. Occurrences of arctic-alpine plants with <10 *sed*aDNA reads in a single PCR replicate are also indicated. Plant taxa inferred to species level based on their biogeographic distribution are marked with an asterisk (*). The x-axis of the incidence matrix refers to the *sed*aDNA sample number, with a decreasing age (towards the present-day) from left to right. The y-axis presents each plant taxon detected using *sed*aDNA sorted according to their median distribution within the *sed*aDNA samples. Plant taxa that are still present within the vegetation of the Polar Urals today are marked with a grey square on the far right-hand side of the incidence matrix; bryophytes are excluded as the present-day biogeographic distribution of many of the identified taxa are poorly resolved. Green shaded boxes indicate the timing of past shrub and forest tree establishment in the vicinity of the lake. Botanical drawings of key plant taxa were created using Adobe Illustrator CC 2018 (https://www.adobe.com/uk/products/illustrator.html#).

A sustained, long-term increase in floristic diversity is observed in the *sed*aDNA record over time (Fig. 2, Supplementary Figs. S1–S3). Of the total 162 plant taxa detected using *sed*aDNA, 70% (114 taxa) were detected within the full-glacial period (ca. 24,000–15,000 cal. years BP), 75% (85 taxa) of which persist into the Holocene period (ca. 11,700–1,300 cal. years BP) along with the addition of 47 new plant taxa not present in the full-glacial period. The majority (87%) of the vascular plant taxa detected within the full-glacial period and 89% of all the vascular plant taxa detected by *sed*aDNA are still found in the vegetation today (Fig. 2, Supplementary Table S2). Thus, much of the present-day flora at Lake Bolshoye Shchuchye was already in place in the full-glacial period; subsequently, there has been underlying continuity in taxonomic composition but also a gradual addition of new plant taxa over time to form the present-day vegetation mosaic.

**Figure 3:**
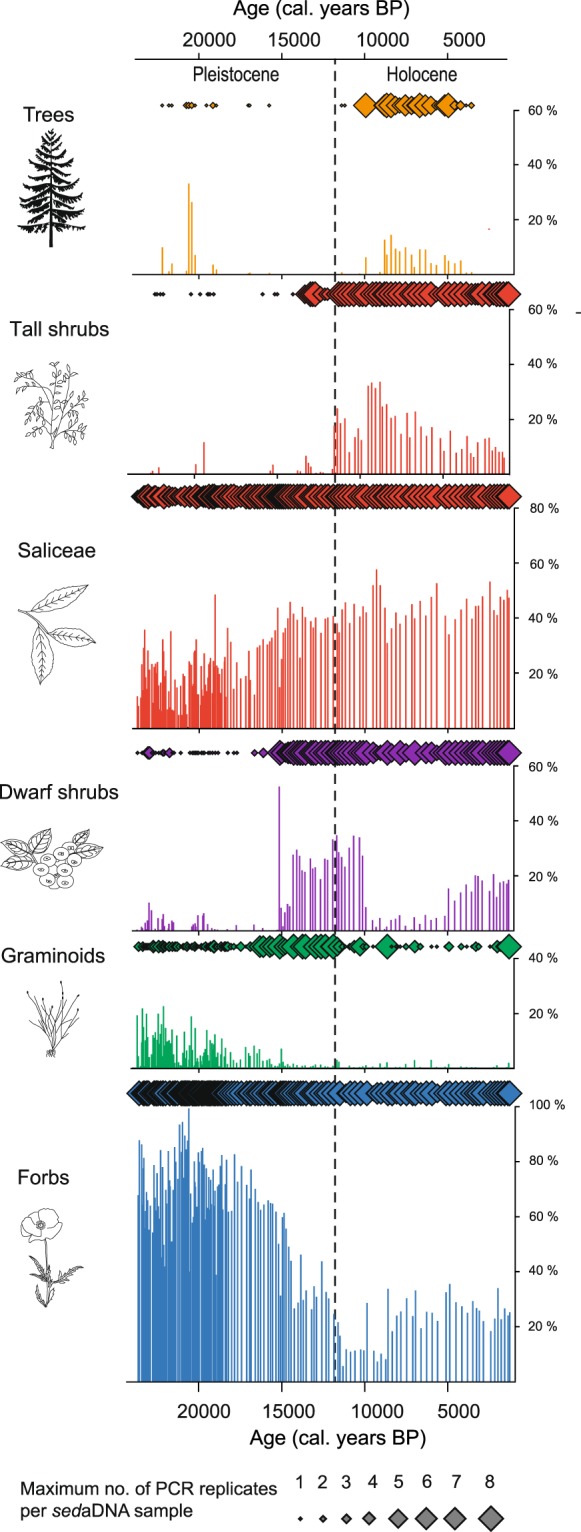
Turnover in plant functional groups over the past 24,000 years. Selected functional groups are presented as a percentage of total *sed*aDNA reads per sample (histogram) and maximum number of PCR replicates (out of eight) per sample (diamond symbols) for the Lake Bolshoye Shchuchye record. Note that the height of the y-axis varies amongst panels. The grey dashed line indicates the Pleistocene-Holocene boundary. Botanical drawings of key plant taxa were created using Adobe Illustrator CC 2018 (https://www.adobe.com/uk/products/illustrator.html#).

Saliceae, a tribe of the willow (Salicaceae) family, is common throughout the record, occurring in nearly all PCR repeats of all samples. It most likely represents dwarf shrubs (e.g. *Salix polaris, S. reptans, S. nummularia*) in the early full-glacial, with the addition of shrub forms (e.g. *Salix lanata*, *S. glauca*, *S. phylicifolia*) in the late-glacial period and potentially tree forms in the Holocene (e.g. *Salix caprea, S. cinerea*). In full-glacial and early late-glacial time (24,000–15,000 cal. years BP), the *sed*aDNA assemblage is characterized by herb-tundra vegetation with a rich diversity of forbs, such as *Papaver, Draba, Bistorta vivipara* and *Saxifraga oppositifolia*, and graminoids, such as *Puccinellia, Festuca* and *Juncus biglumis* (Fig. 3). The mat-forming dwarf shrub *Dryas* becomes more common from 15,000 cal. years BP, followed by a sequential arrival of additional dwarf shrubs and tall shrubs/deciduous trees (e.g. *Betula, Empetrum, Vaccinium* sp.).

The coniferous trees *Picea* sp. and *Larix sibirica* became common elements of the vegetation ca. 9,000–4,000 cal. years BP, alongside many boreal herbs (Fig. 3, Supplementary Figs. S1–S3). Coniferous forest withdrew around 4,000 cal. years BP; the vegetation subsequently reverted to dwarf-shrub tundra with a diverse herb flora, similar to the vegetation mosaic seen in the late-glacial and early Holocene interval, as well as the present-day vegetation mosaic of the Polar Urals.

### Species persistence and floristic richness

**Figure 4:**
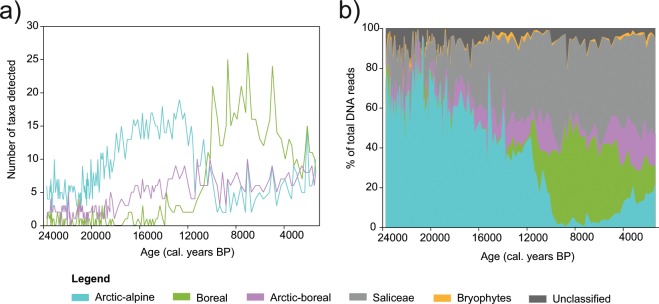
Response of arctic-alpine plants to the past establishment of woody, boreal plants at Lake Bolshoye Shchuchye. (**a**) Floristic diversity over time and (**b**) the proportional abundance of plants detected by *sed*aDNA within each distribution category. For full details of the distribution classifications, see Supplementary Table S2.

We assigned a classification, where possible, to vascular plant taxa detected by *sed*aDNA based on their present-day native distribution (see Methods). In total, 31 taxa were classified as “arctic-alpine”, 49 as “boreal” and 22 as occupying a dual “arctic-boreal” distribution (full details of the classifications are presented in Supplementary Table S2). A steady increase in the diversity of arctic-alpine plants is observed throughout the full-glacial and late-glacial period, peaking around 12,700 cal. years BP before a distinct decline to low values (Fig. 4a). The arctic-boreal taxa, which include widespread genera such as *Empetrum* and *Vaccinium*, show low diversity in the full-glacial period but then increase, with fluctuations similar to the arctic-alpine pattern from around 17,000 cal. years BP. The initial decline in arctic-alpine plants occurs slightly before the main increase in the diversity of boreal taxa, at the time when floristic richness of arctic-boreal taxa is high and *Salix* is abundant. A further decline is seen ca. 10,000 cal. years BP, when the boreal taxa expand and maintain a high diversity until around 4,500 cal. years BP, with four prominent peaks visible in the Holocene period (Fig. 4a). Overall, the proportion of DNA reads of the three distribution categories (Fig. 4b) show similar trends of floristic diversity, with the exception that arctic-alpines reached their maximum proportion (>98% of total DNA reads) around 20,000 cal. years BP, considerably earlier than their maximum diversity was reached.

We investigated the response of individual arctic-alpine plant taxa to the Holocene establishment of dwarf shrub and tall shrub/ trees (e.g. *Dryas octopetala, Vaccinium* sp., *Empetrum nigrum, Betula, Alnus*) and later forest trees (e.g. *Larix sibirica*, *Picea* sp.) in the lake’s vicinity between ca. 15,000–4,000 cal. years BP (Fig. 2). In total, 32 arctic-alpine plant taxa were detected by *sed*aDNA, of which 31 are detected within the full-glacial interval; *Arabis alpina* was recorded during the Holocene interval only (see Supplementary Table S2). Of the 31 arctic-alpines that are detected within the full-glacial interval, 27 (28 including occurrences with <10 DNA reads) are recorded after dwarf shrubs and tall shrubs/trees began to establish from 15,000 cal. years BP and 15 (21) are recorded in the later period when forest tree taxa established between ca. 9,000–4,000 cal. years BP. Of the 17 arctic-alpines that are not detected in the *sed*aDNA during the period of forest tree establishment, all disappear prior to the establishment of forest tree taxa during the preceding shrub period (Fig. 2). Nevertheless, three (four) of these taxa reappear when coniferous forest withdrew in the more recent period (ca. 4,000–1,300 cal. years BP), and all of the 32 arctic-alpine plant taxa detected in the *sed*aDNA record are found in the local vegetation today.

## Discussion

Several studies have emphasised the advantage of *sed*aDNA for higher taxonomic resolution and richness compared to traditional methods of pollen and plant macrofossils^30,35,47^. The *sed*aDNA record from Lake Bolshoye Shchuchye is taxonomically diverse, even compared with most other *sed*aDNA studies^39,48^; this may be explained by its long temporal record, optimised methods and use of a more complete local reference library. Moreover, the sediment lithology is predominantly fine-grained clays and silts; these tend to be highly suitable for the preservation of DNA since extracellular DNA can bind to the relatively large and charged surface areas of clay colloids^49–51^. The lake has a large (215 km^2^) and topographically complex hydrological catchment with steep slopes, high rates of erosion and runoff and considerable riverine input to the lake^42^, all of which probably contribute to the floristically rich *sed*aDNA record obtained. The *sed*aDNA signal in Lake Bolshoye Shchuchye likely reflects sediment inputs from large source areas that capture a range of plant communities occupying the lake’s hydrologic catchment. Since *sed*aDNA was able to identify many taxa that are often poorly represented in traditional palaeorecords, the Lake Bolshoye Shchuchye record provides a unique insight into the response of the artic-alpine flora to environmental change, including the millennial-scale, phased expansion of woody growth forms in the late-glacial and Holocene.

There was both continuity and change in plant community composition at Lake Bolshoye Shchuchye over the past 24,000 years; the lake’s catchment supported a growing set of plant communities, and species diversity increased over millennia with few taxon losses to the present day. It is possible that the observed pattern of increasing species diversity could be explained by better DNA preservation and thus detection of *sed*aDNA within the uppermost sediments compared to older sediments. Data patterns suggest this is not the case, however. A sustained increase in species diversity is observed until the early to middle Holocene, after which species diversity appears to stabilise (with the exception of four anomalous peaks) towards the most recent samples, with little change that could be attributed to better DNA preservation (Supplementary Fig. S5).

More than two thirds of all recorded plant taxa identified were already in place in the full-glacial period (ca. 24,000–15,000 cal. years), when the study region remained free of extensive ice cover^42,52,53^, and the data provide a rich floristic record of full-glacial vegetation close to local glaciers but outside of the Eurasian Ice Sheet margin, which was situated further to the north^53^. Subsequently, new taxa appeared over time, and there was a shift in the dominance of plant functional groups during a long period of changing and (predominantly) warming climate. While the timing and nature of the general vegetation changes observed since the last glacial period at Lake Bolshoye Shchuchye are comparable to those documented in nearby pollen records from the region^54–56^, our record is longer, well-dated and more complete. Furthermore, the *sed*aDNA permits the identification of a sequential arrival of different dwarf-shrub taxa from 15,000 cal. years BP and the clear identification of larch (*Larix sibirica*) forest in the vicinity of the lake between ca. 9,000 and 4,000 cal. years BP (*Larix* is notorious for its low pollen productivity and underrepresentation in pollen records).

It is well accepted that arctic-alpine and boreal-steppe taxa are primarily sensitive to competition for light (rather than sensitive to heat *per se*), and that they can be shaded out by larger, shrubby growth forms^57–59^. From the *sed*aDNA data, it appears that the stepwise addition of shrub and sub-shrub taxa during the late-glacial may well have led to competition and the reduction of populations of many arctic-alpine taxa, resulting in a reduced diversity of arctic-alpines. Notably, the reduction of arctic-alpine plants occurred prior to the arrival of trees, and changes in plant dominance were well underway by the time of forest establishment. Thus, the current expansion of shrub-tundra^58,60^ may pose a threat to the abundance and diversity of arctic-alpines, especially in homogenous tundra habitats where much of this expansion has been observed.

The timing of forest tree establishment in the vicinity of Lake Bolshoye Shchuchye (~9,000 cal. years BP and continuing until ~4,000 cal. years BP) is concurrent with the known northward treeline expansion into the lowlands of the Polar Urals and surrounding areas, which many authors consider representing warmer summer temperatures^61–64^. During this period, when arctic-alpine taxa would have been most disadvantaged, nearly half of the 31 arctic-alpine taxa were detected, at least in small quantities, which strongly suggests their continuous presence in the catchment from full-glacial times; all 31 are found in the region today.

Pollen and plant macrofossil records seldom show such clear evidence of continuity, because of the swamping effect of Holocene anemophilous pollen at virtually all northern sites and differential preservation of identifiable plant remains^65,66^. A survey of European late-Quaternary pollen data showed no increase in diversity into the Holocene in northern sites, in part probably due to this feature^67^. However, exceptional records with high pollen counts in regions never colonized by trees in the Holocene do demonstrate the local persistence of arctic-alpines from the late-glacial period through the Holocene, for example, that from Hanging Lake in mountains of the northern Yukon^26,68^. Although *sed*aDNA cannot fully resolve all aspects of the arctic-alpine plant community, it can greatly enhance the documentation of species persistence in the face of changing environmental conditions.

Intermittent absences inevitably present an interpretational problem in palaeorecords^22,69^. For the subset of taxa which do show absences during the periods of shrub and/or tree expansion, the possibilities are either that they became locally extinct, or that they were present but their DNA input to the sediment dropped below the threshold for detection, probably as a result of their decreased biomass on the landscape at a time when the biomass of woody taxa expanded. Furthermore, taxa that may have been growing near the lake in full-glacial time most likely shifted their range up slope with increasing warmth and associated competition, removing them from the lake edge and valley floor, which is a prime locality for DNA recruitment to sediment^45^. Thus, as with pollen of entomophilous taxa, but probably not to the same extent, there is likely a limitation in detectability for rare taxa in the *sed*aDNA signal.

A complementary line of evidence used to evaluate the likelihood of species persistence is that of modern genetic diversity patterns, where, generally, regions that have supported a flora over a relatively long period show higher levels of genetic diversity than regions recently recolonised. Genetic diversity shows an increasing gradient from Fennoscandia eastward to unglaciated areas of northern Russia and Siberia. Within this gradient, the Polar Urals are classified as intermediate^70,71^. This suggests a degree of long-term persistence (compared with Fennoscandia) and could reflect the presence of plant communities since the last major deglaciation of the northern segment of the Ural Mountains at ~60,000 cal. years BP^42,52,53^.

The arctic-alpine flora has been identified as one that is exceptionally threatened by projected future climate change^3^; it is also one that is often difficult to track via traditional palaeorecords. The Lake Bolshoye Shchuchye record provides unusually robust empirical evidence of the persistence of an arctic-alpine flora through a long period of environmental change, demonstrating the buffering capacity of a spatially heterogeneous landscape. However, a distinct decline in abundance, which is likely related to biomass, began as soon as woody taxa started to expand, suggesting that in a future warming scenario, local species loss may occur long before tree establishment occurs. These empirical results strongly support the need for conservation awareness of potential biodiversity loss in arctic environments, particularly those with less topographic buffering capacity. We conclude that in the Arctic, as elsewhere, conservation priorities should go beyond protecting individual species or areas that are deemed important today and instead move towards monitoring and protecting areas that have shown to be resilient to past changes and thus tend to harbour high genetic diversity and biodiversity.

## Methods

### Study site

Lake Bolshoye Shchuchye (67°53′24″N, 66°18′36″E) is located at 187 m a.s.l. in the central part of Polar Urals mountain chain, ca. 105 km north-east of the mining town of Vorkuta (Fig. 1). The lake itself is ~13 km long and ~1 km wide and has a maximum water depth of 140 m in the centre of the basin. Seismic reflection profiling revealed that the basin fill contains more than 160 m of acoustically laminated sediments with minimal disturbances from mass movements^40,42^. The lake has a catchment area of 215 km^2^, with a deltaic inlet at its northern shore caused by inflow of the River Pyriatanyu and an outlet along its southern shore draining into the Bolshoye Shchuchya River, a tributary of the Ob River. The lake is surrounded by steep-sided valley slopes and exposed rock faces with high mountain peaks reaching 500–1,100 m a.s.l. at its north-western shore, intersected by valleys.

Present-day climate conditions are characterised as cold and continental, with a mean summer temperature (June-July-August) of 7 °C at the Bolshaya Khadata station^72^, located 25 km to the south of Lake Bolshoye Shchuchye at 260 m a.s.l. The lake lies within arctic bioclimatic sub-zone E (Shrub tundra), which is characterised by mean July temperatures of 10–12 °C^73^. The catchment vegetation mosaic comprises dwarf shrub-tundra with patchy thickets of *Alnus viridis* growing on south-facing slopes up to an elevation of 300 m a.s.l. At the higher elevations, the vegetation is discontinuous with exposed rocky surfaces supporting alpine grass and forb communities. The lake is situated close to the northern distributional limit of *Larix sibirica*, with isolated trees observed a few kilometres to the southeast of the lake.

Lake Bolshoye Shchuchye is considered to have been formed by glacial erosion during repeated past glaciations, following weaknesses along ancient NW-SE striking faults^42^. The lake and its catchment are located well outside the maximum extent of the Barents-Kara Ice Sheet during the LGM ca. 25,000–17,000 cal. years BP and the region is thought to have remained ice-free for at least the last 50,000–60,000 cal. years BP^42,52,53^. During time periods around 90,000 cal. years BP (MIS 5b) and 70,000–60,000 cal. years BP (MIS 4), large ice sheets formed over the Barents-Kara Sea region, creating major ice-dammed lakes that flooded the adjacent lowlands on both sides of the Ural Mountains chain^56,74^. It remains controversial whether large ice caps formed over the Polar Urals, the northernmost part of the mountain chain, during this time^75^ but it is clear that sizeable outlet glaciers from mountain valleys reached the foothills of the Polar Urals ca. 60,000 cal. years BP. In contrast to previous glaciations, the Barents-Kara Ice Sheet did not reach the northern rim of the mainland during the LGM and almost the entire Arctic seaboard of Russia to the east of the Arkhangelsk region remained ice-free during this glaciation^74^. Cosmogenic (^10^Be) exposure dating revealed that the small cirque glaciers that exist today within the Polar Urals were only slightly larger during the LGM than today^42,76^.

### Sediment core retrieval

Six separate cores were retrieved from Lake Bolshoye Shchuchye, full details of which are provided by an earlier accompanying paper^42^. A 24-m long core (number 506–48) retrieved in July 2009 from the southern end of the lake (67^o^51′22.248″N, 66^o^21′30.096″E) is the focus for this study. The core was retrieved with a UWITEC piston corer using a combination of 2-m long by 10-cm diameter PVC sample tubes for most sections and 2-m long by 9-cm diameter steel tubes for the deepest sections. Since collection in July 2009, the core sections remained unopened and sealed within light-proof cylinders in a cold storage facility until they were opened and split longitudinally in the winter of 2014, when subsamples for radiocarbon dating were taken. The core sections were sealed and placed back into cold storage until subsampling for *sed*aDNA analyses was undertaken in the winter of 2015. A detailed sedimentological description and the core chronology (Supplementary Fig. S4), which is based on a series of AMS radiocarbon dates supported by a sequence of annual laminations (varves), is presented in two earlier publications^41,42^.

### Radiocarbon dating and sampling of the sediment core

In total, 26 samples of terrestrial macrofossils were radiocarbon (^14^C) dated with Accelerator Mass Spectrometry (AMS) at the Poznań Radiocarbon Laboratory of the Adam Mickiewicz University, Poland. All radiocarbon ages were calibrated according to the terrestrial IntCal13 curve^77^ using the online Calib program^78^. Subsampling of the core was undertaken at ca. 15-cm resolution in a laminar flow cabinet within a clean laboratory within the Centre for Geobiology and Microbiology at the Department of Earth Science, University of Bergen, Norway, using sterile tools, full bodysuit, facemask, and gloves. Subsampling was undertaken in the presence of subsampling controls (open water samples) in order to detect potential lab aerial contamination. Following the protocol described by Parducci *et al*.^36^ the outer 10 mm of sediment was avoided and an ~20 g subsample was retrieved from inside the freshly exposed centre only.

### DNA extraction, amplification, library preparation and sequencing

DNA extraction, PCR amplification, PCR product pooling and purification, and sequencing followed the protocols of Alsos *et al*.^35^ and Clarke *et al*.^31^ unless otherwise stated. DNA was extracted from 153 sediment subsamples within the dedicated ancient DNA facility at Tromsø University Museum (TMU), Norway. In addition, DNA was extracted from 17 negative extraction, nine negative PCR and nine negative water (subsampling) controls, which contained no sediment and were used to monitor for contamination. Aliquots of DNA extracts were then shipped to the Laboratoire d’ÉCologie Alpine (LECA, Université Grenoble Alpes, France) for metabarcoding. Each DNA extract and negative extraction control was independently amplified using uniquely-tagged generic primers that amplify the *trn*L P6 loop of the plant chloroplast genome^79^ in eight PCR replicates to increase confidence in the results and improve the chance of detecting taxa with small quantities of template in the DNA extracts^80^. Pooled and cleaned PCR products were then converted to four Illumina-compatible amplicon libraries using the single-indexed, PCR-free MetaFast method (FASTERIS SA, Switzerland). These libraries were then sequenced on the Illumina HiSeq-2500 platform for 2 × 125 cycles at FASTERIS.

### DNA sequence analysis and taxonomic assignments

Next-generation sequence data were filtered using the OBITools software package (Boyer *et al*., 2016; http://metabarcoding.org/obitools/doc/index.html) following the protocol and criteria defined by Alsos *et al*.^35^. Taxonomic assignments were performed by first matching sequences against a reference library of 2445 sequences comprising 815 artic^32^ and 835 boreal vascular plants^29^, in addition to 455 arctic/boreal bryophytes^33^. Sequences were then matched to a second reference library generated after running *ecopcr* on the global EMBL database (release r117).

In order to minimise any erroneous taxonomic assignments, only taxa with a 98% match, or greater, to a reference sequence were retained. A minimum threshold of at least 10 reads per PCR replicate was used, except in the case of the arctic-alpine plant taxa (see Fig. 2) where <10 reads per PCR replicate occurrences are highlighted. We further removed sequences that displayed higher average frequency in the negative subsampling (water), extraction or PCR controls than in the lake sediment samples in which they were present. Identified taxa were compared to the circumpolar flora^81^, Pan Arctic Flora Checklist^82^ and flora of the Polar Urals^83–85^. Sequences with <100% match to a reference sequence and/or those assigned to taxa that are not present in northern Russia today were carefully crosschecked against the NCBI BLAST database for multiple or alternative taxonomic assignments (http://www.ncbi.nlm.nih.gov/blast/). Taxonomy follows the Pan Arctic Flora Checklist^82^, if present.

### Distributional classification of taxa

Where possible, we assigned a classification of either “arctic-alpine”, “boreal” or “arctic-boreal” to each vascular plant taxon identified by *sed*aDNA based on their present-day native distribution^81,86^. Plant taxa classified as “arctic-alpine” had two-thirds or more of their main distributional area located north of the forest limit for boreal tree taxa *Pinus, Picea, Larix* and *Betula*. Those classified as “boreal” had two-thirds or more of their distributional area located within the boreal forest limits. Finally, those assigned an “intermediate” classification occupied an area where approximately half of their main distributional area was located within each biome. For those taxa which were identified to genus or family level only by *sed*aDNA, a classification was given based on the distribution of all likely species within the identified genus or family which are present within northern Russia today. Full details of the classifications, including likely species of which the classification is based upon, are presented in Supplementary Table S2. Bryophytes were excluded from the analysis due to the lack of sufficient information on their present-day distribution.

To determine the degree of species persistence over time and long-term continuity of individual plant taxa identified by *sed*aDNA, an assessment of their present-day occurrence within the local vegetation mosaic of Lake Bolshoye Shchuchye’s hydrologic catchment was performed based on data collected by L. Morozova and colleagues during the years 2000, 2001 and 2006 for a period of 46 days in total. Botanical data were collected systematically along topographical/ecological transects from rivers/lakes up to the top of a ridge or mountain. In addition, botanical information was retrieved from several sources^83–85^.

## Supplementary information

Supplementary Information (Figures S1 to S6)

Supplementary Tables S1 and S2

## Data Availability

The forward and reverse reads for the four amplicon libraries analysed from Lake Bolshoye Shchuchye, along with the used primer and tag sequences per sample are available within the DRYAD database at 10.5061/dryad.jdfn2z378.
