# Supplementary material for: Persistence of arctic-alpine flora during 24,000 years of environmental change in the Polar Urals
Source: Sci Rep. 2019 Dec 23;9:19613. doi: 10.1038/s41598-019-55989-9 (PMC6927971; doi:10.1038/s41598-019-55989-9)
Supplement: Supplementary file 1 — Supplementary Information (Figures S1 to S6) [file 41598_2019_55989_MOESM1_ESM.pdf]

Supplementary Information

Title: Persistence of arctic-alpine flora during 24,000 years of environmental change in the Polar Urals

Clarke, C.L.*, Edwards, M.E., Gielly, L., Ehrich, D., Hughes, P.D.M., Morozova, L.M., Haflidason, H., Mangerud, J., Svendsen, J.I. and Alsos, I.G.

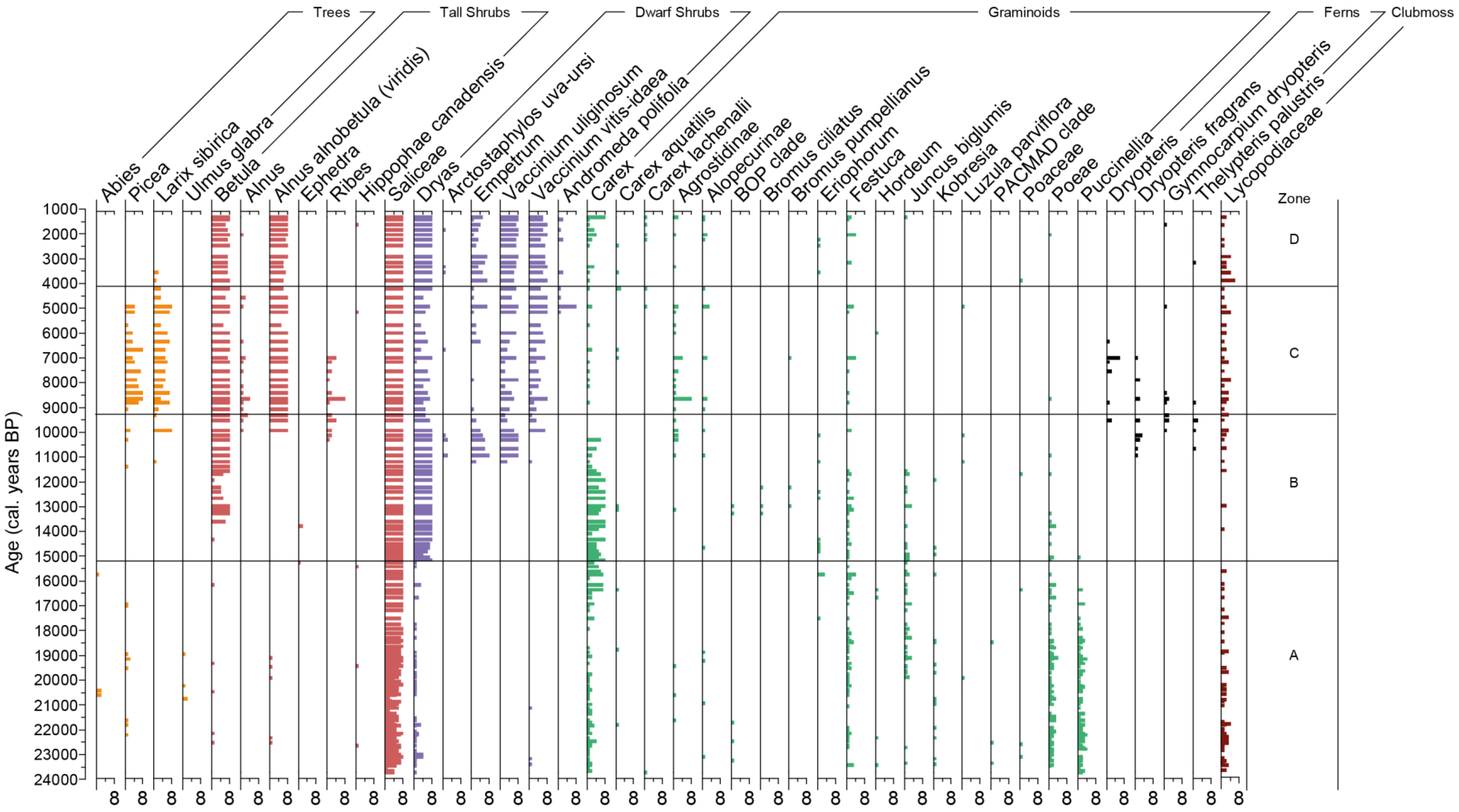

Supplementary Figure S1:

Woody, graminoid, fern and clubmoss taxa detected by *seda*DNA at Lake Bolshoye Shchuchye. The x-axis refers to number of PCR replicates (out of eight) a taxon was detected within per sample.

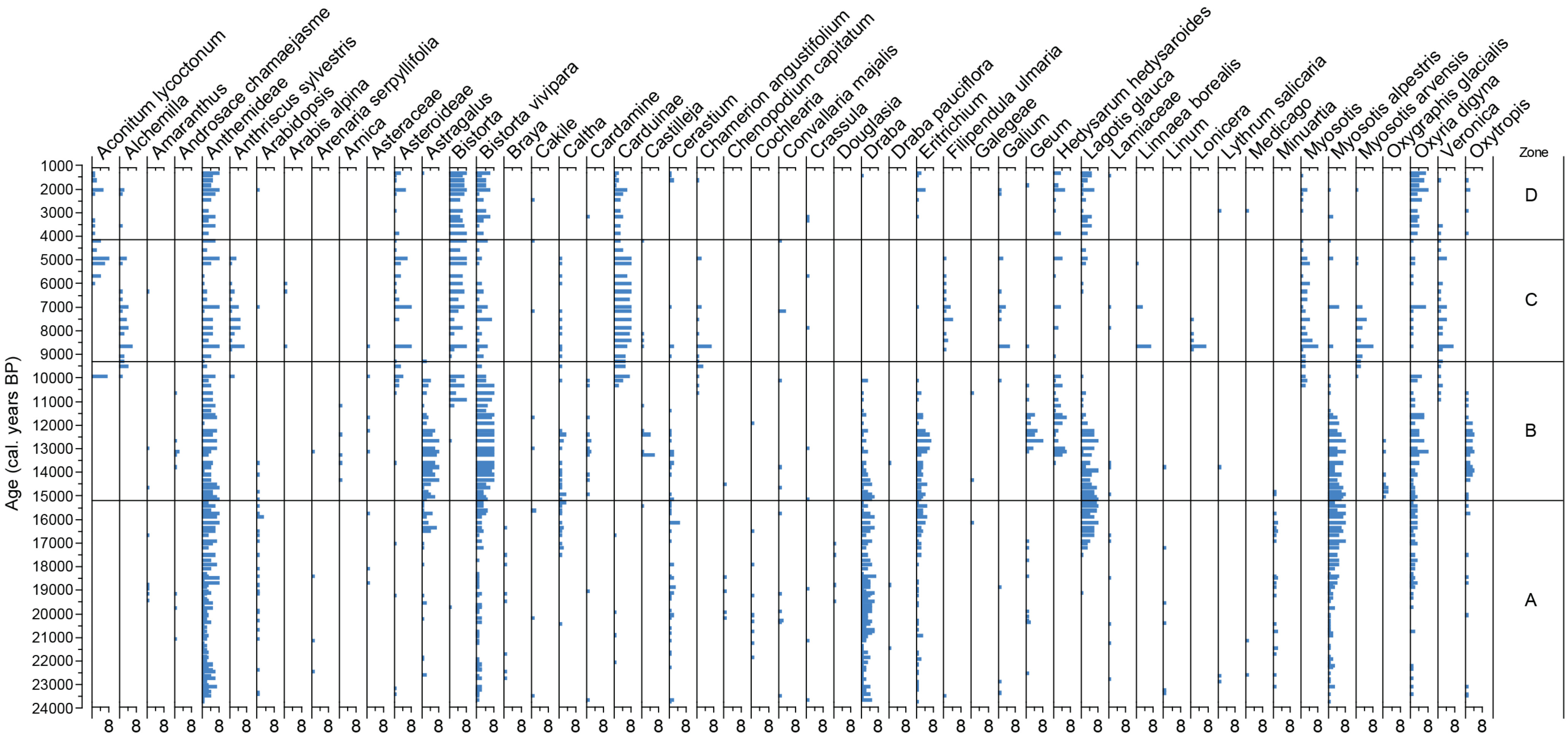

Supplementary Figure S2:

Forb taxa detected by *sed*aDNA at Lake Bolshoye Shchuchye. The x-axis refers to number of PCR replicates (out of eight) a taxon was detected within per sample.

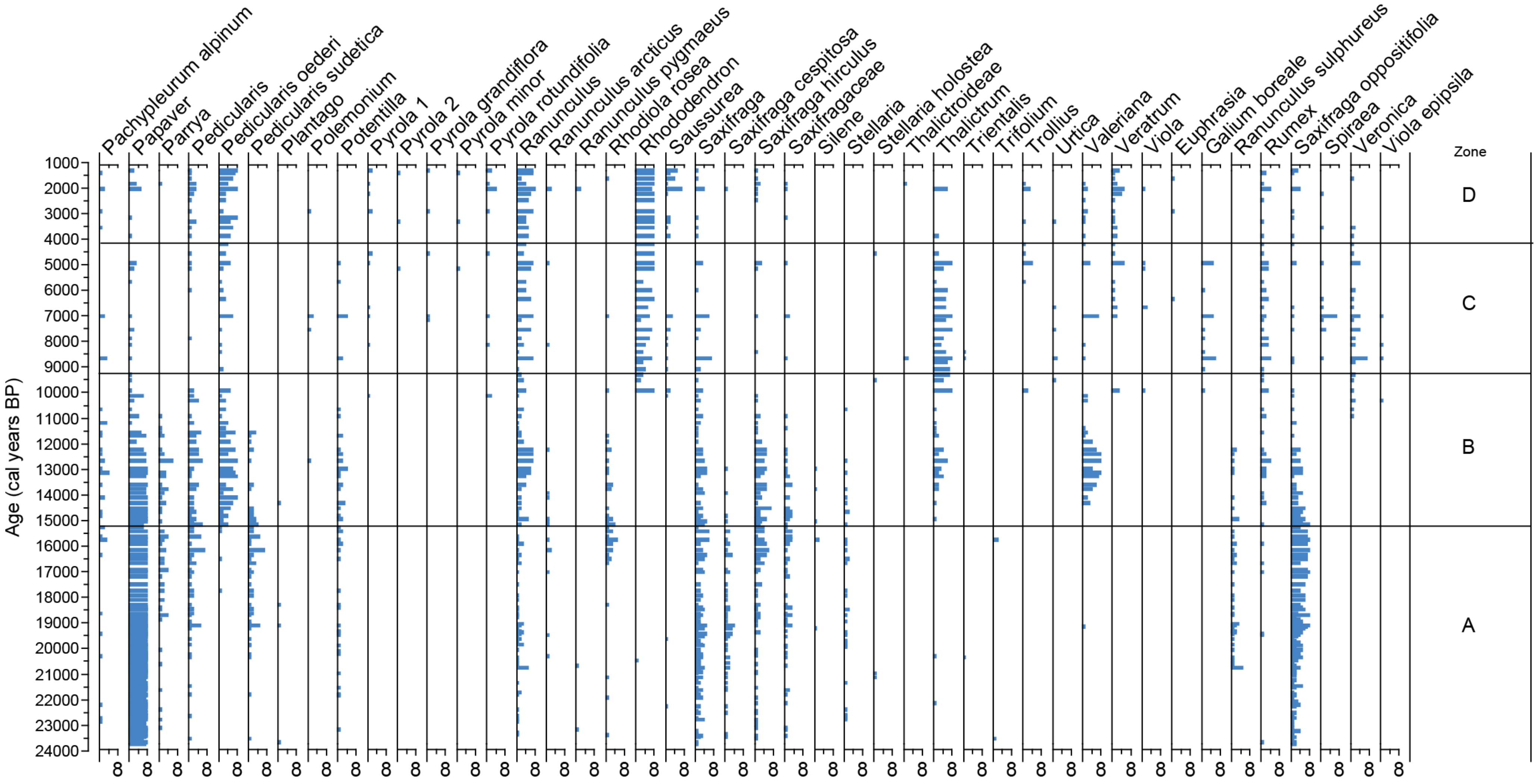

Supplementary Figure S2 cont.:

Forb taxa detected by *sed*aDNA at Lake Bolshoye Shchuchye. The x-axis refers to number of PCR replicates (out of eight) a taxon was detected within per sample.

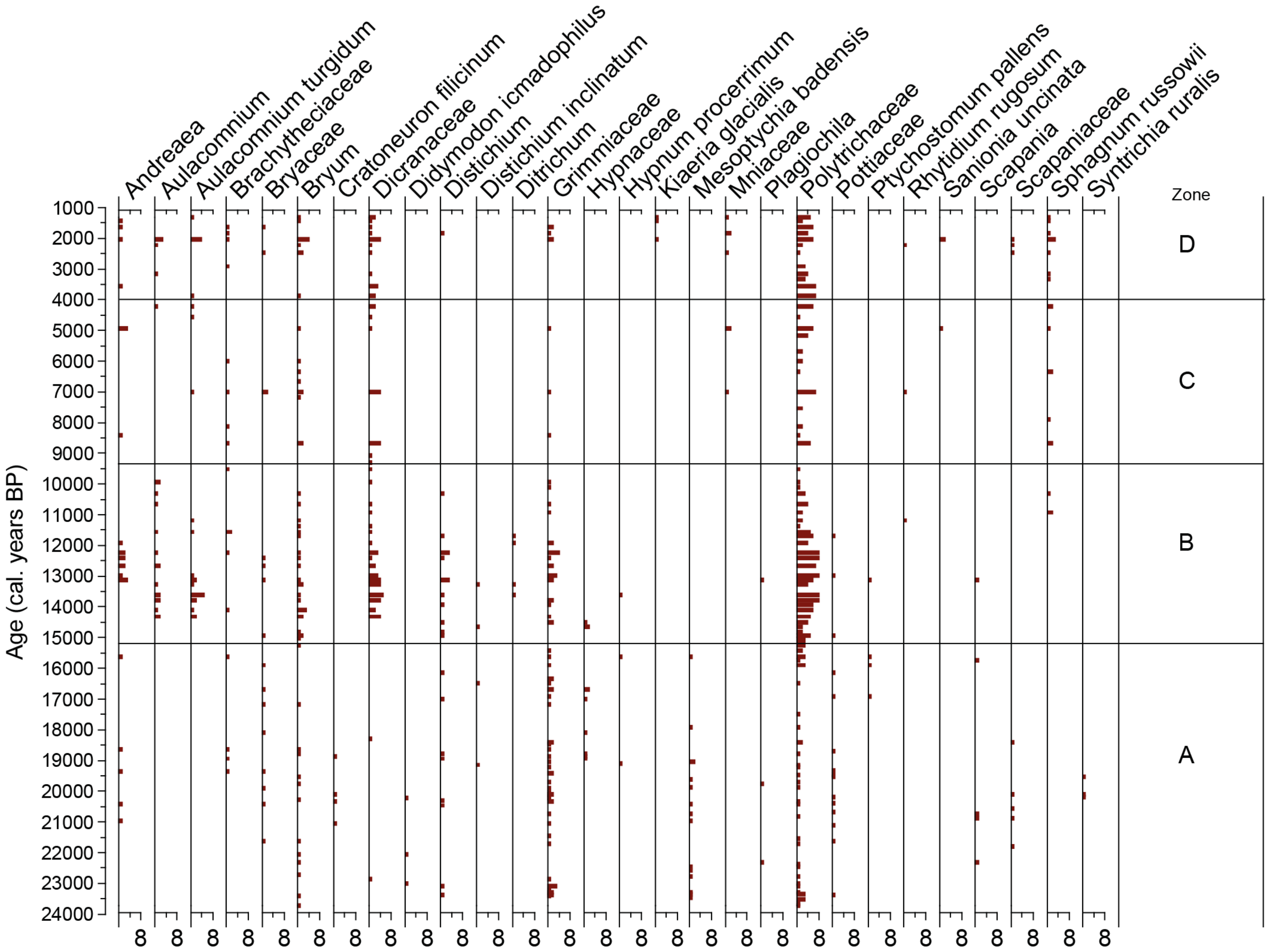

Supplementary Figure S3:

Bryophyte taxa detected by *sed*aDNA at Lake Bolshoye Shchuchye. The x-axis refers to number of PCR replicates (out of eight) a taxon was detected within per sample.

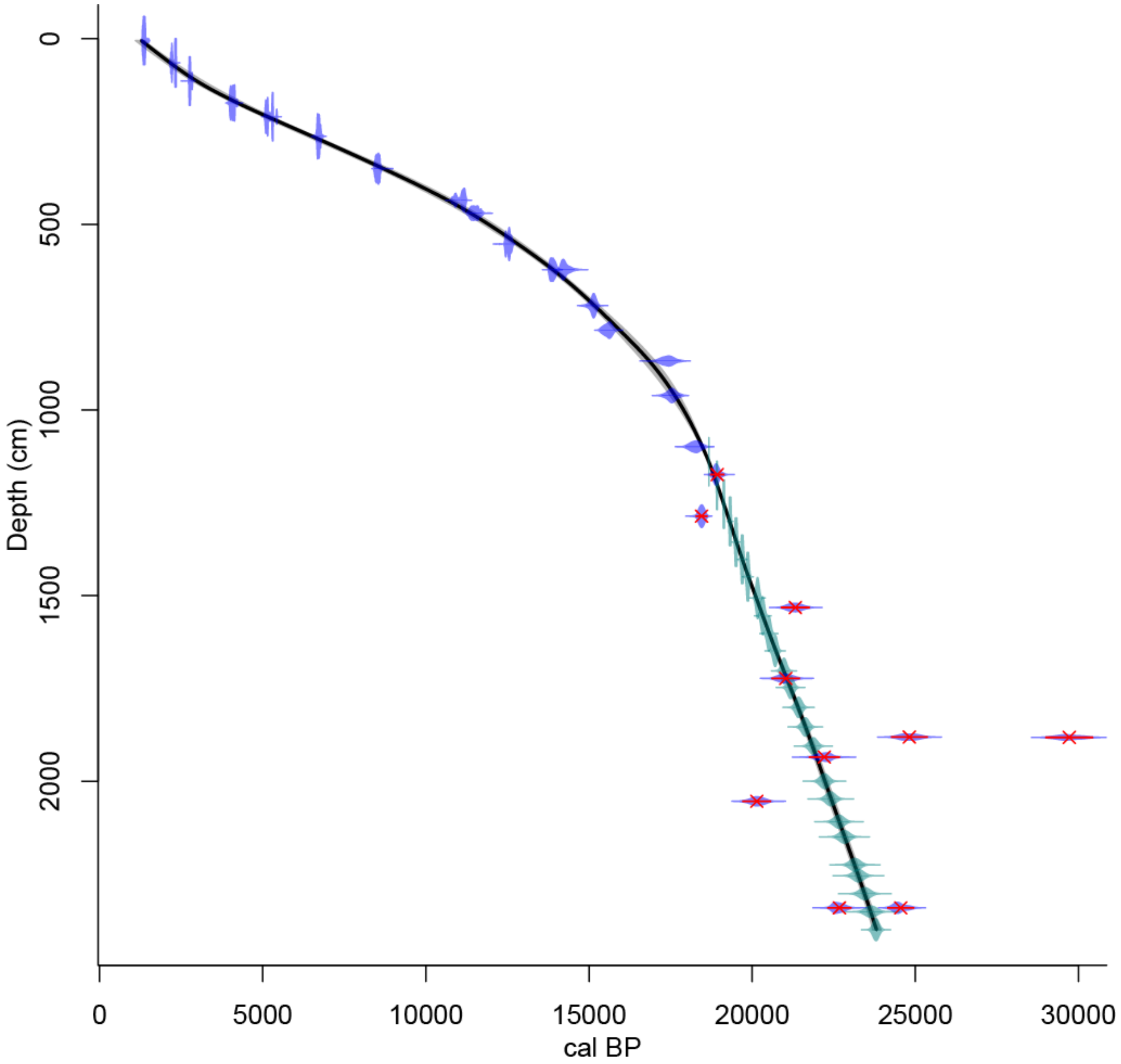

Supplementary Figure S4:

The age-depth relationship for core no. 506-48 from Lake Bolshoye Shchuchye, Polar Urals. Small grey-shaded area shows the 95 % probability interval. The green marks show chronology retrieved from counting of annual laminations (varves) from 11.4 m depth to the base of the core. The varve chronology is anchored to 18,679 ± 128 cal. years BP (95 % confidence interval) and gives a basal age of 23,813 ± 306 cal. years BP (see Regnéll *et al*., 2019). The blue marks show the probability distribution of AMS radiocarbon ages; outliers are marked with red crosses (Svendsen *et al*., 2019).

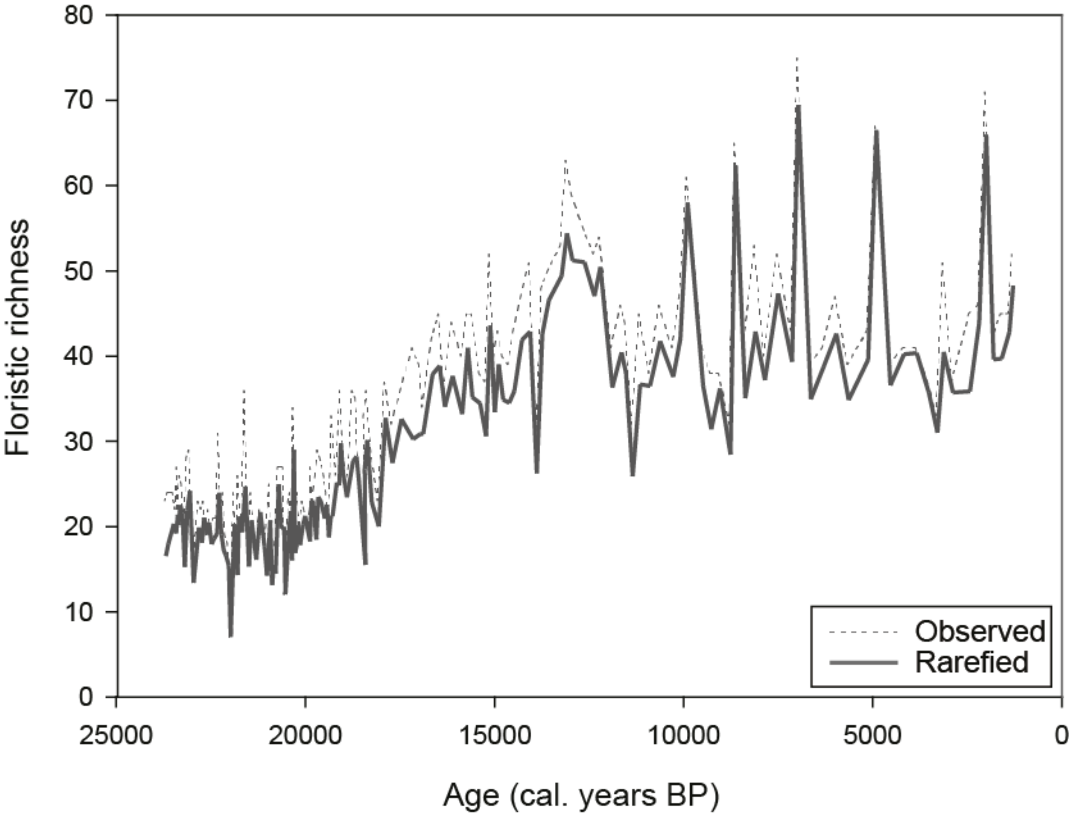

Supplementary Figure S5:

Observed (pre-rarefaction) and rarefied (post-rarefaction) richness based on *sed*aDNA at Lake Bolshoye Shchuchye over time. Rarefaction analysis was performed in the Vegan (Oksanen et al., 2017) package for R (R Core Team, 2017) using the minimum DNA read count.

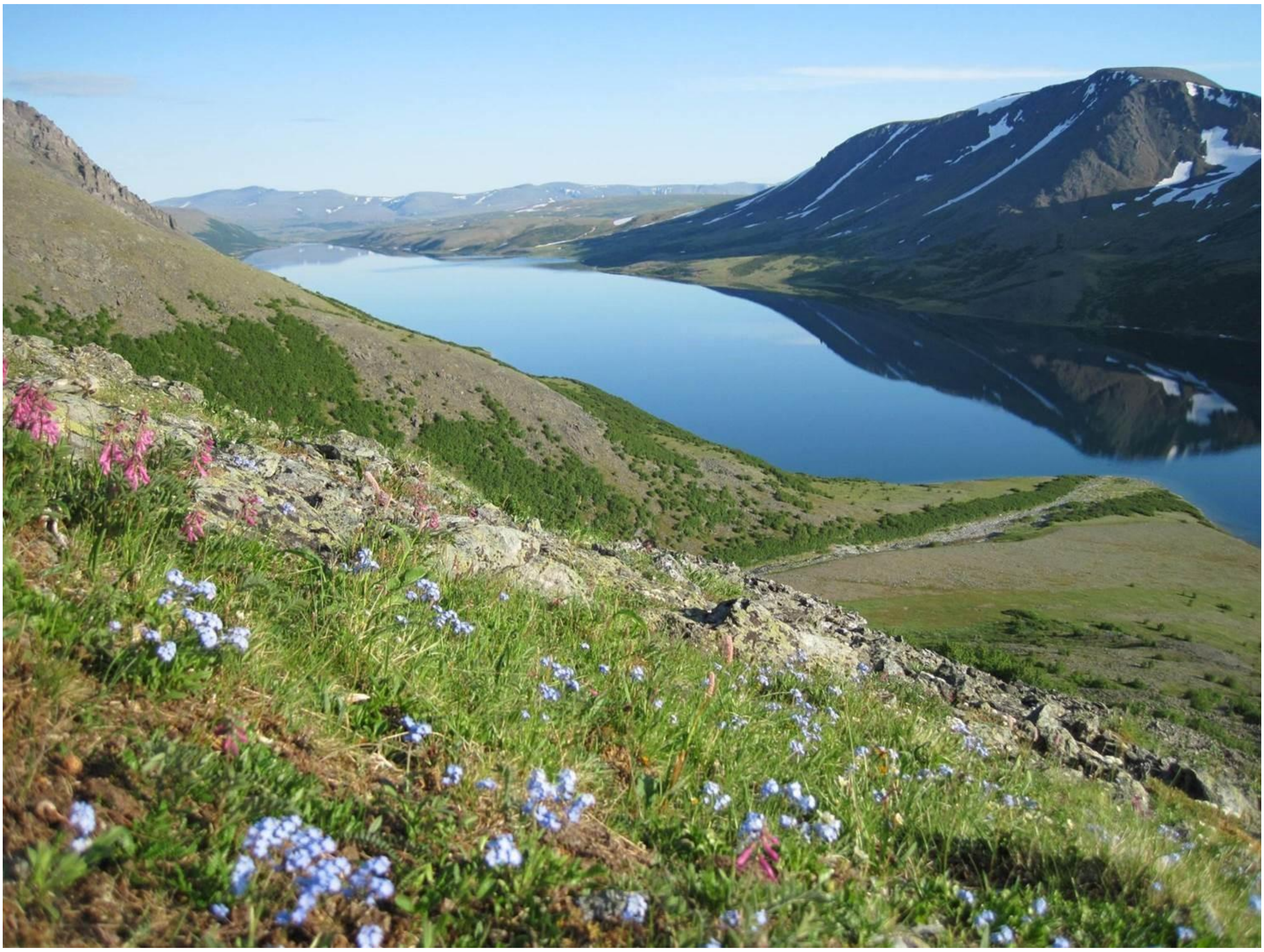

Supplementary Figure S6:

Overview photograph of Lake Bolshoye Shchuchye taken on 14 July 2009 from the northern end of the lake. The dark green bushes growing on the alluvial fan and on the slope are green alder (*Alnus viridis*). Note the alluvial fan in the foreground. Arctic-alpine forbs such as *Myosotis* and *Chamerion* occupy fellfield habitats at the highest elevations.

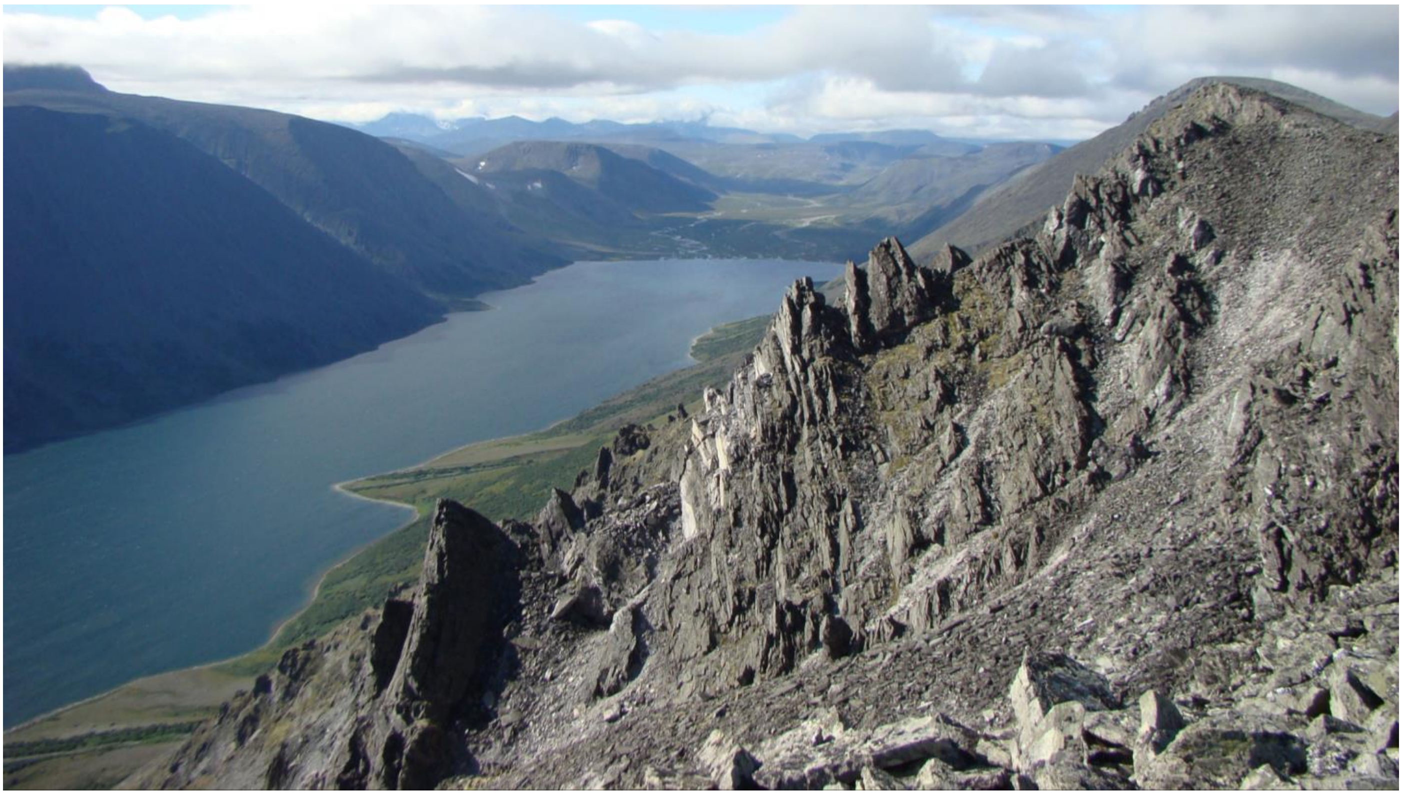

Supplementary Figure S7:

Overview photograph detailing the topographic characteristics of Lake Bolshoye Shchuchye's catchment taken from the highest elevations on the eastern side of the lake looking towards the deltaic inlet on the northern shore. Mountain peaks in the catchment range between 50 and 1,100 m a.s.l.

References

Oksanen, J., Blanchet, F. G., Friendly, M., Kindt, R., Legendre, P., McGlinn, D., Minchin, P. R., O'Hara, R. B., Simpson, G. L., Solymos, P. M., Stevens, H. H., Szoecs, E. and Wagner, H. Vegan: community ecology package. Version 2.4-2. R package. Available at: https://CRAN.R-project.org/package5vegan (2017)

R Core Team 2017: R: A language and environment for statistical computing. R Foundation for Statistical Computing, Vienna. Available at: http://www.R-project.org/ (2017)

Regnéll, C., Haflidason, H., Mangerud, J. and Svendsen, J. . Glacial and climate history of the last 24 000 years in the Polar Ural Mountains, Arctic Russia, inferred from partly varved lake sediments. Boreas 48, 432–443 (2019).

Svendsen, J. I., Færseth, L.M.B., Gyllencreutz, R., Haflidason, H., Henriksen, M., Hovland, M.N., Lohne, Ø., Mangerud, J., Nazarov, D., Regnéll, C. and Schaefer, J.M. Glacial and environmental changes during the last 60,000 years in the Polar Ural Mountains, Arctic Russia, inferred from a high resolution lake record and observations from adjacent areas. *Boreas* 48, 407–431 (2019).
